# Supplementary material for: Synthesis and Configurational Character Study of Novel Structural Isomers Based on Pyrene–Imidazole
Source: Molecules. 2019 Jun 20;24(12):2293. doi: 10.3390/molecules24122293 (PMC6630678; doi:10.3390/molecules24122293)
Supplement: Supplementary file 1 [file molecules-24-02293-s001.pdf]

Synthesis and Configurational Character Study of Novel Structural Isomers Based on Pyrene-Imidazole

Yu-Long Liu [1,2], Liu Yang [1], You-Quan Guo [3], Guo-Qiang Xu [1], Bin Qu [1,2] and Ying Fu [1,*]

[1] Department of Applied Chemistry, College of Science, Northeast Agricultural University, Harbin 150030, China; liuyulong@neau.edu.cn (Y.-L.L.); yangliu@neau.edu.cn (L.Y.); xuguoqiang@neau.edu.cn (G.-Q.X.); qubin@neau.edu.cn (B.Q.)

[2] CAS Key Laboratory of Renewable Energy, Guangzhou Institute of Energy Conversion, Guangzhou 510640, China;

[3] Heilongjiang Vocational College of Biology Science and Technology, Harbin 150025, China; feilong50123146@126.com

* Correspondence: fuying@neau.edu.cn; Tel.: +86-451-5519-0070

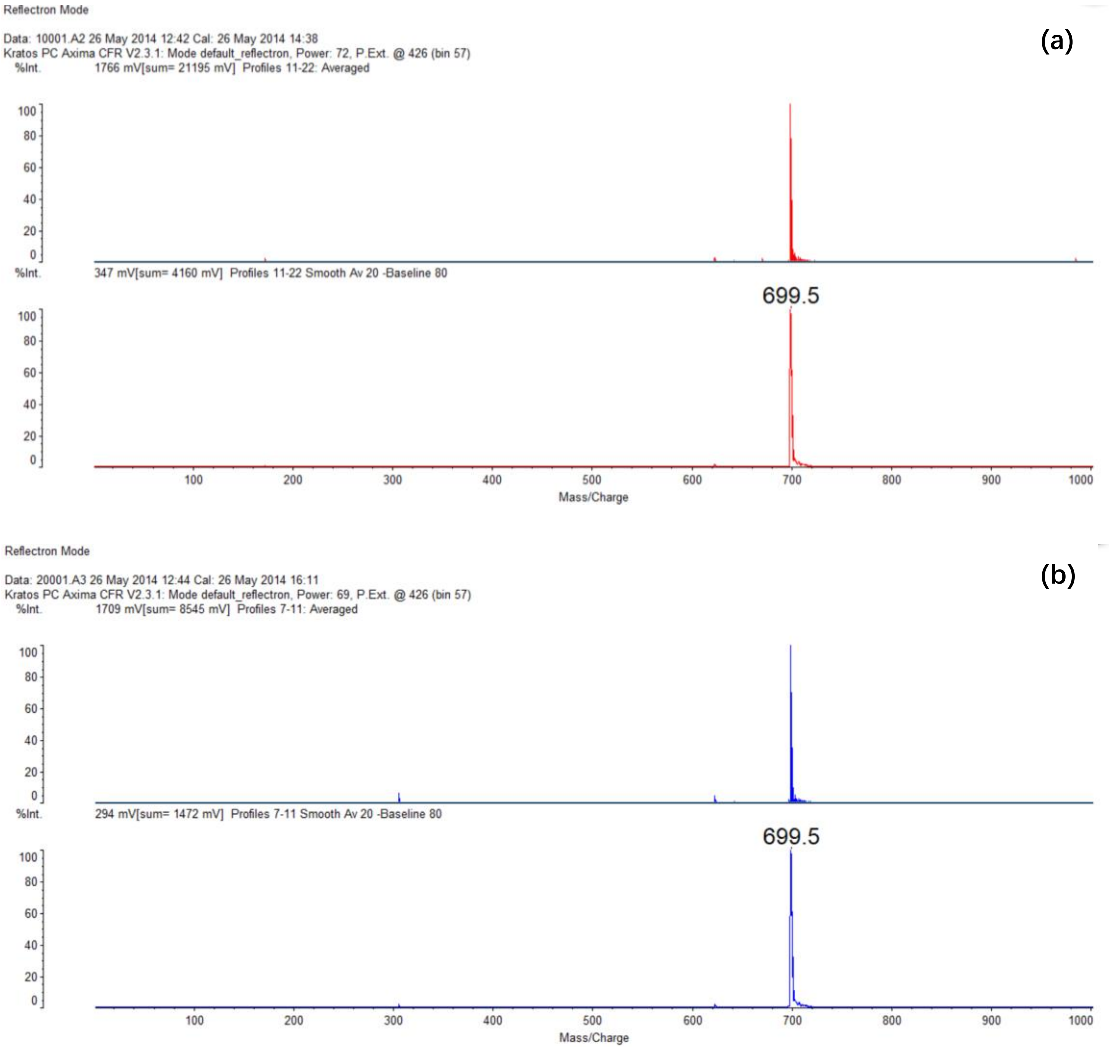

Figure S1. Mass Spectrum (M+H$^+$) of *anti*-tbu-PyDPI (a) and *syn*-tbu-PyDPI (b).

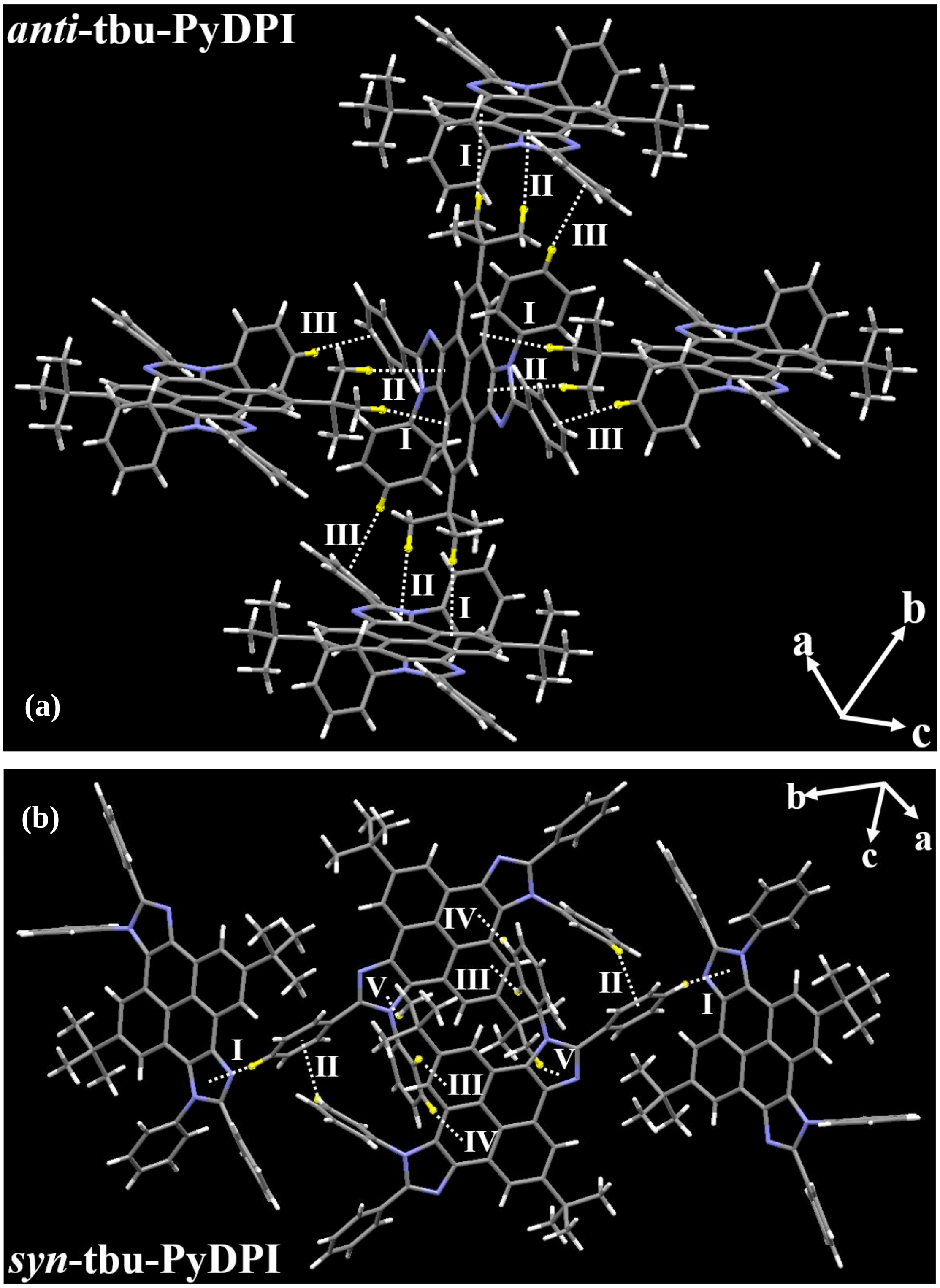

Figure S2. Packing form and the C-H•••π intermolecular interactions in crystal of *anti*/*syn*-tbu-PyDPI.

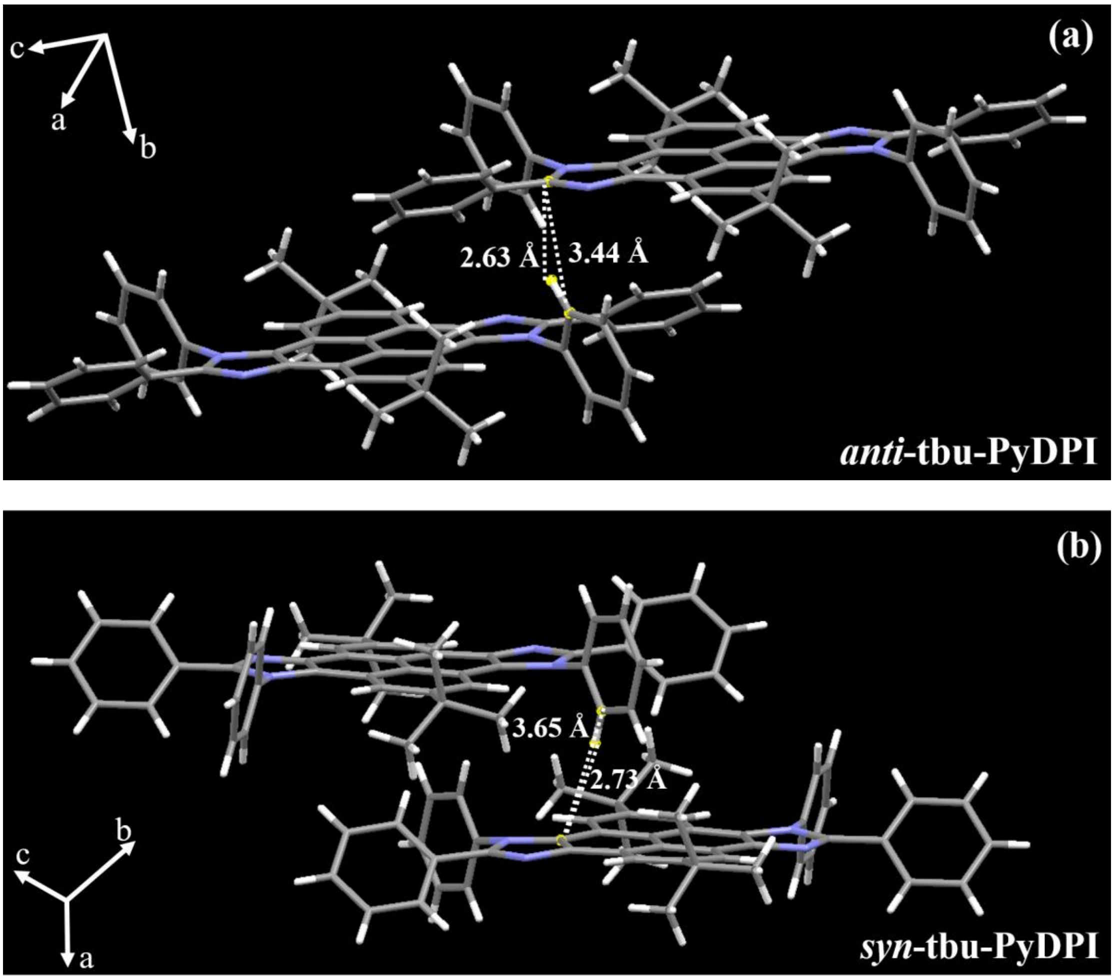

Figure S3. Short CH(sp^2)···π interactions with phenyl groups at N atoms of *anti*/*syn*-tbu-PyDPI.

Table S1. Crystal data and structure refinements of isomers.

Compound	*anti*-tbu-PyDPI	*syn*-tbu-PyDPI
empirical formula	$C_{50}H_{42}N_4$	$C_{50}H_{42}N_4$
formula wt	698.88	698.88
T, K	296(2)	153(2)
crystal system	Monoclinic	Monoclinic
space group	P 2(1)/c	P 2(1)/c
a, Å	10.116(2)	11.756(2)
b, Å	16.062(3)	26.760(5)
c, Å	11.970(2)	13.272(3)
α, deg	90	90
β, deg	104.72(3)	110.23(3)
γ, deg	90	90
V, Å^3	1881.2(6)	3917.7(14)
Z	2	4
density, Mg/m^3	1.234	1.185
Absorption coefficient, mm^{-1}	0.072	0.069
θ range, deg	3.09-25.00	3.04-25.00
no. of reflections collected	14579	27659
no. of unique reflections	3307	6875
R(int)	0.1546	0.0863
Good-of-fit on F^2	1.006	0.963
$R1$ [$I > 2\sigma(I)$]	0.0829	0.0578
$wR2$ [$I > 2\sigma(I)$]	0.1436	0.1473
$R1$ (all data)	0.1826	0.1126
